# The SoxE factor Sox9 is selectively expressed in indirect pathway striatal projection neurons and regulates synaptogenesis

**DOI:** 10.1016/j.fmre.2024.02.019

**Published:** 2024-04-02

**Authors:** Xiaolei Song, Xin Li, Xingru Pan, Hongkun Yang, Kun Wang, Tao Yang, Liyao Guo, Xiaoming Xin, Weidong Le, Rongliang Guo, Zhejun Xu

**Affiliations:** a Shanghai University of Medicine & Health Sciences Affiliated Zhoupu Hospital, Shanghai 200237, China; b Center for Clinical and Translational Medicine, Shanghai University of Medicine and Health Sciences, Shanghai 200237, China; c The Affiliated Hospital and the School of Landscape and Ecological Engineering, Hebei University of Engineering, Handan 056000, China; d Institute for Translational Brain Research, State Key Laboratory of Medical Neurobiology, MOE Frontiers Center for Brain Science, Fudan University, Shanghai 200433, China; e Dental Disease Prevention and Treatment Clinic of Minhang District, Shanghai 201100, China; f Jitang College of North China University of Science and Technology, Tangshan 063000, China; g The Affiliated Hospital and the Medical College, Hebei University of Engineering, Handan 056000, China; h Key Laboratory of Birth Defects, Children’s Hospital of Fudan University, State Key Laboratory of Medical Neurobiology and MOE Frontiers Center for Brain Science, Institutes of Brain Science, Fudan University, Shanghai 200433, China

**Keywords:** Sox9, Drd2-MSN, Drd1-MSN, LGE, Striatal development, Synaptogenesis

## Abstract

Striatum, as the largest structure of the basal ganglia, serves as a center for information transmission and is critical for motor function and reward perception. However, the genetic mechanisms underlying its development require further exploration. Here, we found that *Sox9*, traditionally recognized as a glial marker, is uniquely expressed in striatal medium spiny neurons (MSNs), especially in *Drd2*-expressing indirect pathway MSNs (D2-MSNs). Intriguingly, *Sox9* expression in the striatum, which is conserved in humans, is a dynamic process. It maintains a high level during the perinatal stage, and exhibits low expression levels or vanishes at the embryonic and postnatal stages, respectively. The peak period of *Sox9* expression coincides with the transition from neurogenesis to synaptogenesis. Importantly, gene regulatory network analysis and gain-of-function experiments confirmed *Sox9* is strongly correlated with synaptogenesis. Moreover, we identified that *Sox9* regulates synaptogenesis by repressing *Foxp2*, a well-known synapse regulator. Furthermore, we demonstrated that the biased expression pattern of *Sox9* in D2-MSNs is, at least in part, regulated by another SoxE family member *Sox8*, which is specifically expressed in *Drd1*-expressing direct pathway MSNs (D1-MSNs). Taken together, our findings reveal a new marker of D2-MSNs and identify its distinctive function in striatal development.

## Graphical abstract

**Figure:**
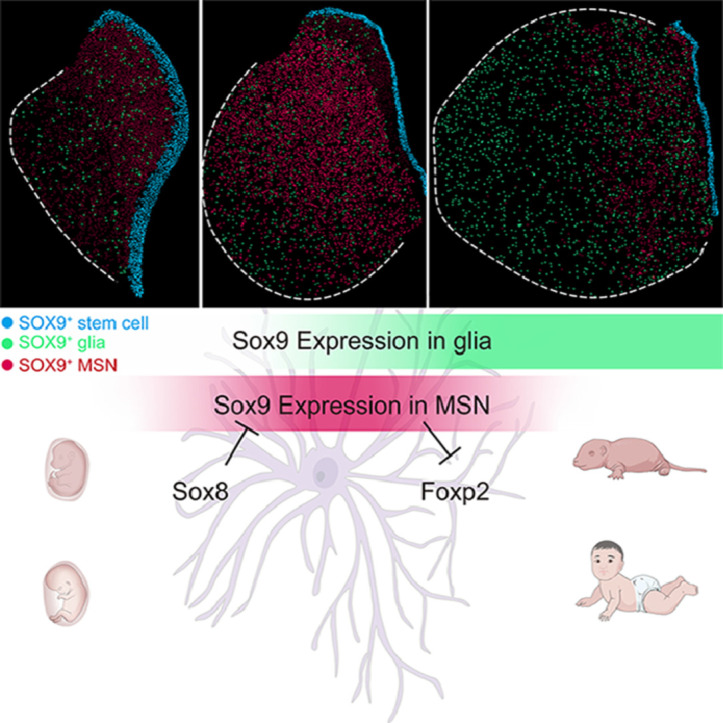

## 1 Introduction

The striatum is a crucial structural and functional component of the basal ganglia. Its abnormal development significantly contributes to brain dysfunctions, such as schizophrenia, Huntington’s disease, Parkinson’s disease, and autism [[1], [2], [3]]. As an information transmission center, the striatum receives inputs from the thalamus and nearly all cortical areas, transmitting signals to output nuclei [1]. Striatal projection neurons, also known as MSNs due to their morphological features, comprise 90%−95% of striatal neurons [4]. MSNs are subdivided into two types based on the differential expression of dopamine receptor subtypes: dopamine type I (D1) and type II (D2) receptor expression MSNs [5]. D1-MSNs directly project to the internal segment of the globus pallidus (GPi) and the substantia nigra pars reticulata (SNr), functioning as direct pathway projection neurons. Conversely, D2-MSNs project to the lateral globus pallidus (GPe) and then project to the SN, thus named as indirect pathway projection neurons [1].

The striatum originates from the lateral ganglionic eminence (LGE) [6]. Numerous transcription factors have been identified to regulate LGE development and determine the fate specification of the two MSN subtypes. *Zfp503* is a crucial transcription factor in regulating the fate transition between D1 and D2 MSNs; and its deletion significantly increases the production of D2-MSNs while reciprocally generating few D1-MSNs [7,8]. *Sp9* expressed in LGE progenitors, and specifically in postmitotic D2-MSNs, regulates their generation and survival [6]. Moreover, *Sp8* and *Sp9* double knockout mice exhibit an almost complete absence of D2-MSNs with minimal effect on D1-MSN development [4]. *Six3*, a downstream target of *Sp9* in regulating D2-MSN development, promotes D2-MSN differentiation [4,9]. The distinct projection features and expression profiles of the two MSN subtypes are essential for maintaining striatal homeostasis [1,5]. Any abnormal development in striatal neurons can disrupt this balance and potentially induce disease, making it crucial to understand the gene-regulated developmental processes of the striatum.

*Sox9*, traditionally known as an astrocyte marker, is highly enriched in the ventricular zone (VZ) of the LGE and is necessary for maintaining the multipotentiality of neural stem cells [[10], [11], [12], [13], [14], [15], [16]]. In this study, we demonstrate that *Sox9* is expressed not only in glial cells but also in the striatal projection neuron lineage. Analysis of single cell RNA sequencing (scRNA-seq) data from various developmental stages, including embryonic and postnatal periods, revealed significantly higher levels of *Sox9* expression in D2-MSNs compared to that in D1-MSNs. Furthermore, scRNA-seq data from the human LGE during gestational weeks (GW) 9 to18 show a conserved and consistent expression pattern of *Sox9* in D2-MSNs. Additionally, we found that this particular expression pattern of *Sox9* in D2-MSNs contributes to synaptogenesis by regulating *Foxp2*. Taken together, our findings reveal a previously unidentified striatum-specific function of *Sox9*, enhancing our understanding of striatal development and related diseases.

## 2 Materials and methods

### 2.1 Animals

All experimental procedures involving mice were approved by the Animal Ethics Committee of Fudan University (Ethical approval number: 20220228-136) and were performed in accordance with the National Institutes of Health Guide for the Care and Use of Laboratory Animals. The mice utilized in the experiments were maintained on a mixed genetic background of C57BL/6J and ICR strains. The day of vaginal plug detection was defined as E0.5, and the day of birth was considered as P0.

### 2.2 Immunohistochemistry

Postnatal mice were anesthetized and perfused with PBS, followed by fixation in 4% paraformaldehyde (PFA), before the brain tissues were collected. Both embryonic and postnatal brains were post-fixed in 4% PFA overnight, and then transferred to 30% sucrose for dehydration at 4 °C. The brain tissues were embedded in Optimum Cutting Temperature (OCT) compound for sectioning.

For immunohistochemistry, brain slides were collected and rinsed with 0.05 M TBS to remove any residual OCT. They were then treated with 0.5% Triton X-100 for 30 mins at room temperature (RT) to facilitate the following antigen-antibody reaction. Slices were incubated in 5% donkey serum for 2 h at RT to block the unspecific targets. Primary antibodies were then applied and the slices were stored at 4 °C overnight. Following this incubation, the slices were washed with 0.05 M TBS to remove the primary antibodies. Then secondary antibodies were included and incubated for 2 h in the dark at RT. After washing off the secondary antibodies, the nucleus was stained with DAPI, and the slices were coverslipped with a mounting medium for staining evaluation.

The primary antibodies used in this study are as follows: rabbit anti-ALDH1L1 (Abcam, ab10712968), rat anti-BCL11B (Abcam, ab18465), goat anti-FOXP2 (Santa Cruz, sc-21069), rabbit anti-GFP (Aves Labs, GFP-1020), rabbit anti-ISL1 (Abcam, ab20670), rabbit anti-SOX9 (Abcam, ab185966), goat anti-SOX9 (R&D System, AF3075), guinea pig anti-SOX10 (Oasis Biofarm, OB-PGP001), and rabbit anti-SP9 (gift from professor Zhengang Yang).

### 2.3 scRNA-seq analysis

All scRNA-seq data were loaded into Seurat (v4.3.0) for analysis. Due to variability in data quality across studies, we applied study-specific filters to the expression matrices based on appropriate criteria. For E14.5 mouse LGE data, cells with a detected gene number ranging from 200 to 4,000 and a mitochondrial gene percentage below 15% were selected for further analysis. For E18.5 mouse ventral telencephalon dataset, cells with a gene detection range between 1,000 and 6,000 and a mitochondrial gene percentage below 20% were included for subsequent analyses.

After filtering, data normalization and scaling were performed using the NormalizeData and ScaleData functions. The top 2,000 variable genes were identified using the FindVariableFeatures function, followed by running PCA, finding neighbors, and clustering cells using the RunPCA, FindNeighbors and FindClusters functions. We also applied the CellCycleScoring function to mitigate cell cycle effects on the data. Clusters were visualized using the uniform manifold approximation and projection (UMAP) method. Differentially expressed genes (DEGs) were identified using the FindAllMarkers function, facilitating cluster annotation. The Harmony package was also used to remove batch effects in the GW09-GW18 human ganglionic eminence data. For developmental trajectory analysis, the Monocle3 package (v1.3.1) was utilized to model and construct the single-cell developmental trajectories of the mouse E18.5 LGE lineage.

### 2.4 Gene Ontology

Gene Ontology (GO) enrichment analysis was conducted using the clusterProfiler package (v4.2.2). The GO terms were further analyzed and clustered using Metascape [17], with results visualized through Cytoscape [18] and the ggplot2 package (v3.4.2).

### 2.5 SCENIC analysis, regulon analysis

The SCENIC pipeline facilitated the calculation of cell-type-specific regulatory networks, focusing on postmitotic MSN lineage cells [19]. Initially, GRN Boost identified gene and transcription factor co-expression modules from data sourced from the RcisTarget database, yielding over 2,000 transcription factor-gene interactions in the MSN dataset. These co-expression modules were subjected to motif enrichment analysis to discard genes without a regulatory element of the transcription factor, resulting in more reliable transcription factor-gene linkage and the generation of regulons. The regulon activity was scored by AUCell with the default settings. Cells with specific regulon enrichment were identified based on the distribution of AUC scores. Regulon analysis was conducted using the SCENIC package (v1.1.2–01), adhering to default parameters. The activity of regulons, averaged across cells with identical annotations, was depicted in a heatmap to highlight the most significantly enriched regulons per annotation.

### 2.6 Quantification of dendritic spine density

Embryos were electroporated at E15 with Sox9 overexpression and dsRed reporter vector. At P16, brains were collected by rapid removal, immersion, and washing in cold oxygenated artificial cerebrospinal fluid (ACSF). Then, brains were sliced into coronal sections at 250 µm and incubated in a chamber continuously aerated with 95% O_2_/5% CO_2_ at 32 °C-34 °C. GFP^+^ dsRed^+^ cells were filled with Alexa Fluor-488 for 5 mins, followed by morphological reconstruction using two-photon laser scanning microscopy with a 25x water immersion objective (zoom x3). Cells without GFP or dsRed expression were randomly chosen and filled with Alexa Fluor-488 for 5 mins as controls in the contralateral striatum. Images were obtained at a wavelength of 920 nm and image stacks were set with a 0.7 µm step size. Spines in the proximal parts of secondary dendrites were counted for each neuron after z-stack projection. The average spine number per 10 µm is presented.

### 2.7 Plasmid construction

To construct the overexpression vectors, the coding sequences of *Sox9* and *Sox8* were cloned downstream of the CAG promoter. H2B-GFP was used to label these overexpressed proteins, and the internal ribosome entry sites (IRES) sequence was cloned to be located between the target protein and the H2B-GFP reporter. Moreover, the β-globin polyA sequence was used to terminate transcription and was positioned at the end of the H2B-GFP reporter gene. The In-Fusion cloning kit (NovoProtein) was used to construct these vectors.

### 2.8 *In utero* electroporation

The IUE procedure was performed according to previous studies [20] using 7-mm platinum electrodes and a BTX830 electroporator. Briefly, 4% isoflurane and 2% isoflurane were used to induce and maintain anesthesia in E14.5 pregnant mice, respectively. Plasmid solutions (1.5–2 mg/ml DNA plus 0.05% Fast Green) were injected into the lateral ventricle of embryos. Five pulses (lasting 50 ms each) with an interval of 950 ms were applied and a 65 V voltage was used to electroporate DNA into the cells at E14.

### 2.9 Data source, imaging, and statistical analysis

In this study, mouse and human scRNA-seq data were downloaded from the public Gene Expression Omnibus (GEO) database (E14.5 mouse LGE GSE202551 [21], E18.5 mouse ventral telencephalon GSE174392 [22], GW9-GW18 human ganglionic eminence and GW18 human LGE GSE135827 [23]). Images were captured using an Olympus BX51 microscope or an Olympus FV3000 confocal microscope system. All images were merged, cropped, and optimized equally using Adobe Photoshop CC and Adobe Illustrator. All quantification results were presented as mean ± SEM. An unpaired Student’s *t*-test was used to determine statistical significance. *P*-values less than 0.05 were considered significant. *, *P* < 0.05; **, *P* < 0.01; ***, *P* < 0.001.

## 3 Results

### 3.1 Expression patterning of *Sox9* in the early developing striatum

**Fig. 1:**
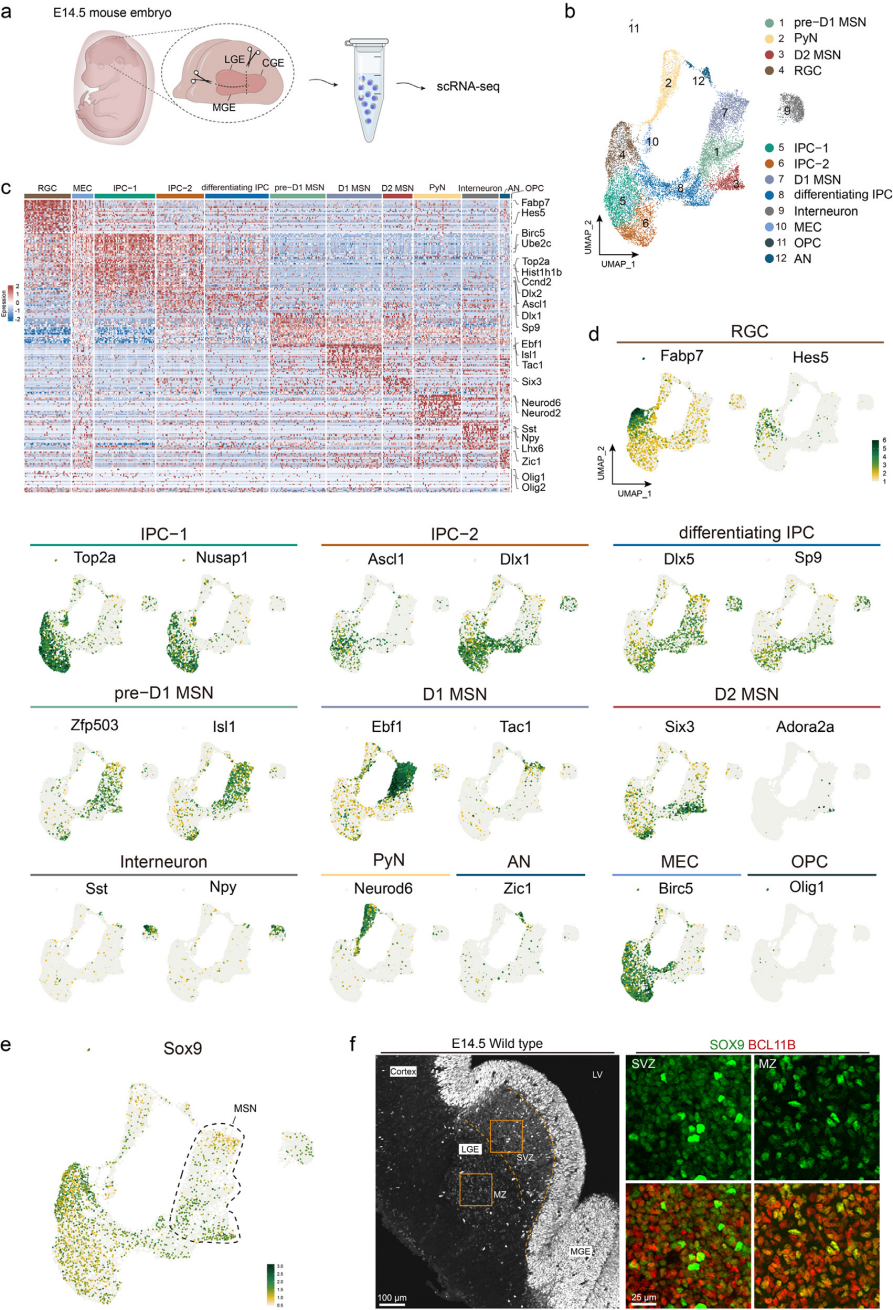
**The expression pattern of *Sox9* in the mouse LGE at E14.5.** (a) Schematic workflow overview of E14.5 mouse LGE scRNA-seq. (b) UMAP plot shows 12 clusters of E14.5 mouse LGE colored by cell-type as identified by DEGs and typical marker genes. (c) Heatmap shows relative expression of genes enriched in 12 different cell types. Typical cell-type marker genes are labelled. (d) UMAP plot shows the expression profiles of typical marker genes in each cluster. Each dot colored by gene expression level, represents an individual cell. (e) UMAP plot shows *Sox9* expression levels in different cell types of E14.5 mouse LGE. Dots within the dashed line represent MSNs. (f) Representative SOX9 staining (white) is shown in the left panel. Dashed lines indicate the borders of VZ, SVZ and MZ. Local magnification of SVZ and MZ fields, stained for SOX9 and BCL11B, are shown in the right panel, respectively.

To assess the expression pattern of *Sox9* in the developing LGE, we first examined the scRNA-seq data of mouse embryonic LGE at E14.5, which corresponds to the early stage of striatal MSN emergence [21]. By trimming the raw data, 14,849 cells were obtained for subsequent analysis (Fig. 1a). Using unsupervised clustering to differentiate cell expression difference and applyingUMAP for dimensionality reduction, we identified 12 clusters with cell-type-specific markers (Fig. 1b, c). Each cluster exhibited a relatively balanced number of features and counts (Fig. S1). The transcriptional signatures of the 12 specific cell types were revealed (Fig. 1c). Based on proviouly reported cell-type-specific markers, we identified 7 common cell types in the LGE, radial glial cell (RGC, e.g. markers *Fabp7* and *Hes5*), intermediate progenitor-1 (IPC-1, e.g. markers *Top2a* and *Nusap1*), IPC-2 (e.g. markers *Ascl1* and *Dlx1*), differentiating IPC (e.g. markers *Dlx5* and *Sp9*), pre-D1 MSN (e.g. markers *Zfp503* and *Isl1*), D1 MSN (e.g. markers *Ebf1* and *Tac1*), and D2 MSN (e.g. markers *Six3* and *Adora2a*) (Fig. 1c, 1d). Additionally, five other clusters were identified: projection pyramidal neuron (PyN e.g. markers *Neurod6* and *Neurod2*), interneuron (e.g. markers *Sst* and *Npy*), mitotic endothelial cell (MEC, e.g. marker *Birc5*), amygdala neuron (AN, e.g. marker *Zic1*), and oligodendrocyte progenitor cell (OPC, e.g. marker *Olig1*) (Fig. 1c, 1d).

In order to investigate the expression of *Sox9* in the early stage of striatal development, we examined its expression in each cell type. We observed a high expression level of *Sox9* in RGCs within the scRNA-seq data in agreement with previous reports [14,15]. Interestingly, *Sox9* was also detected in the IPCs and MSNs, suggesting that, unlike its exclusive expression in glial cells in the cortex, *Sox9* is also expressed in the neuronal lineage in the striatum (Fig. 1e). To confirm this, we performed immunohistochemistry staining for SOX9 at E14.5 mouse embryos. We not only found a high expression level of SOX9 (SOX9^high^) in the VZ, but also observed dense SOX9^+^ cells in the striatal SVZ and MZ (Fig. 1f). It should be noted that, the expression levels of SOX9 were relatively lower (SOX9^low^) in the SVZ and MZ compared with those of SOX9^high^ radial glial cells in the VZ (Fig. 1f). Moreover, most of these SOX9^low^ cells co-expressed BCL11B (Fig. 1f), a pan-MSN marker [24], indicating that Sox9, beyond being a glial marker, is specifically expressed in striatal projection neurons.

### 3.2 Sox9 is selectively expressed in D2-MSNs

To further confirm the above phenotype, we re-analyzed the scRNA-seq data of the E18.5 ventral telencephalon, which contains the LGE, septum, and medial ganglionic eminence (MGE, Supplemental Fig. 2a) [22]. We were interested in determining whether *Sox9* is uniformly expressed in striatal MSNs or predominantly expressed by one of the subtypes. We classified the cells using unsupervised clustering and analyzed the differential gene expression after quality control (Fig. S2b), resulting in 14 major clusters including RGC (e.g. marker *Hes5*), cycling IPC (e.g. marker *Nusap1*), IPC (e.g. marker *Ascl1*), differentiating IPC (e.g. markers *Gadd45g* and *Sp9*), pre-D1 MSN (e.g. marker *Isl1*), D1-MSN (e.g. marker *Ebf1*), D2-MSN (e.g. markers *Six3* and *Adora2a*), dorsal LGE (dLGE) (e.g. marker *Nr2f2*), Septum (e.g. marker *Zic4*), MGE (e.g. marker *Lhx6*), PyN (e.g. marker *Neurod6*), Pericyte (e.g. marker *Rgs5*), microglia (e.g. marker *C1qc*), and endothelial cells (EC, e.g. marker *Tnfrsf19*) (Fig. S2c, 2d).

**Fig. 2:**
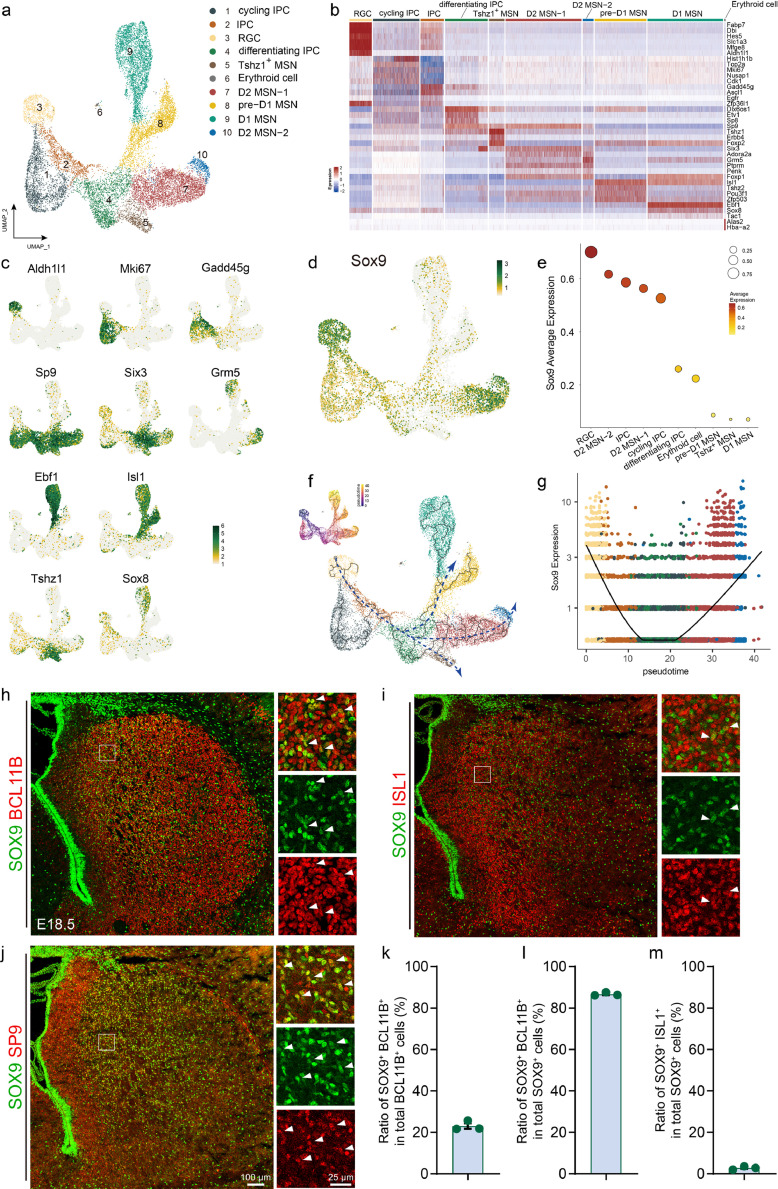
***Sox9* is selectively expressed in D2-MSNs of E18.5 mouse embryo LGE**. (a) UMAP plot shows 10 clusters of E18.5 mouse LGE colored by cell-type as identified by DEGs and typical marker genes. (b) Heatmap shows relative expression of genes enriched in 10 different cell types. (c) UMAP plot shows the expression profiles of typical marker genes in 10 clusters. Each dot colored by gene expression level, represents an individual cell. (d) *Sox9* expression levels in different cell types of E18.5 mouse LGE visualized by UMAP. (e) The cell expression ratio (represented by circle size) and average expression level (represented by color) of *Sox9* in 10 clusters. (f) Trajectory based on pseudotime analysis is depicted in dash line with arrows. (g) *Sox9* expression level along with pseudotime. Each dot represents an individual cell, and colors represent the specific cluster. (h-j) Immunostaining for SOX9 and BCL11B, SOX9 and ISL1, SOX9 and SP9, of E18.5 wild-type mouse LGE. Local magnifications are shown in the right panel. Arrows indicate the co-expressed cells. (k) Quantification of the ratio of SOX9^+^ BCL11B^+^ in total BCL11B^+^ cells. (l) Quantification of the ratio of SOX9^+^ BCL11B^+^ in total SOX9^+^ cells. (m) Quantification of the ratio of SOX9^+^ ISL1^+^ in total ISL1^+^ cells.

As our focus was on LGE development and this dataset contained multiple domains, it was necessary for us to isolate the cells originating from the LGE, particularly the progenitor cells. We utilized unsupervised clustering to categorize the ventral telencephalon progenitor cells into 6 transcriptionally distinct clusters, each exhibiting different gene expression profiles (Fig. S2e, 2f). The expression of *Nkx2.1* characterized MGE progenitor cells, while the expression of *Zic* genes, for example *Zic2*, defined progenitors in the septum (Fig. S2f, 2g). In addition, we also identified a small cluster of OPC characterized by the expression of *Pdgfra* and *Sox10* (Fig. S2f, 2g). Since LGE/striatum is the largest structure of the ventral telencephalon at E18.5, the remaining cells with high expression levels of *Gsx2, Six3*, and *Sp9* were defined as LGE progenitor cells (Fig. S2f, 2g). Furthermore, the cells with high expression levels of *Hes5, Fabp7*, and *Mfeg8* were characterized as radial glial cells (Fig. S2f). We then merged the LGE stem cells and progenitors with the post-mitotic MSNs and performed unsupervised clustering to classify these LGE cells (Fig. 2a). A total of 10 clusters with distinct gene expression profiles were obtained (Fig. 2b, 2c), such as RGC (e.g. marker *Aldh1l1*), IPC (e.g. marker *Gadd45g*), cycling IPC (e.g. marker *Mki67*), differentiating IPC (e.g. marker *Sp9*), Tshz1^+^ MSN (e.g. marker *Tshz1*), Erythroid cell (e.g. marker *Alas2*), D2-MSN-1 (e.g. marker *Six3*), D2 MSN-2 (e.g. marker *Grm5*), pre-D1 MSN (e.g. marker *Isl1*), and D1-MSN (e.g. marker *Ebf1*). Within this filtered dataset, we found that, similar to the E14.5 scRNA-seq data, *Sox9* was enriched in radial glial cells, intermediate progenitor cells, and especially D2 MSNs (Fig. 2d). The average expression level was calculated, showing that *Sox9* was expressed at a relatively high level in D2-MSNs, but not D1-MSNs, in the LGE at E18.5 (Fig. 2e). Further investigation through pseudotime analysis identified three cell trajectories (D1-MSN, D2-MSN, and Tshz1^+^ MSN) diverging from common progenitor cells (Fig. 2f). Importantly, we found that *Sox9* expression is initially highly expressed in radial glial cells, then downregulated in progenitor cells, and finally upregulated in post-mitotic D2-MSNs along the developmental trajectory (Fig. 2g). This result suggests that apart from functioning in radial glial cells, *Sox9* may also play an important role in the development of D2-MSNs.

We then immunostained SOX9 with BCL11B and observed that SOX9^+^ cells were predominantly located in the dorsal-medial part of the striatum and approximately 1/4 striatal MSNs co-expressed SOX9 at this stage (Fig. 2h, 2k), indicating that SOX9 is expressed in neonatal MSNs. Additionally, the vast majority of SOX9^+^ cells co-expressed with BCL11B (Fig. 2h, 2l), indicating that, similar to E14.5, SOX9 in the striatum is also as a marker for striatal projection neurons in addition to being expressed in the glial lineage. Moreover, immunostaining for SOX9 with ISL1 showed that only a very small subpopulation of SOX9^+^ cells expressed ISL1 (Fig. 2i, 2m). However, immunostaining SOX9 with SP9, a specific D2-MSN marker [6], showed a noticeable abundance of double positive cells for SOX9 and SP9 in the striatal MZ (Fig. 2j), indicating that SOX9 is selectively expressed in D2-MSNs, in agreement with the above scRNA-seq results.

### 3.3 The expression of *Sox9* in mouse striatal MSNs is conserved in humans

**Fig. 3:**
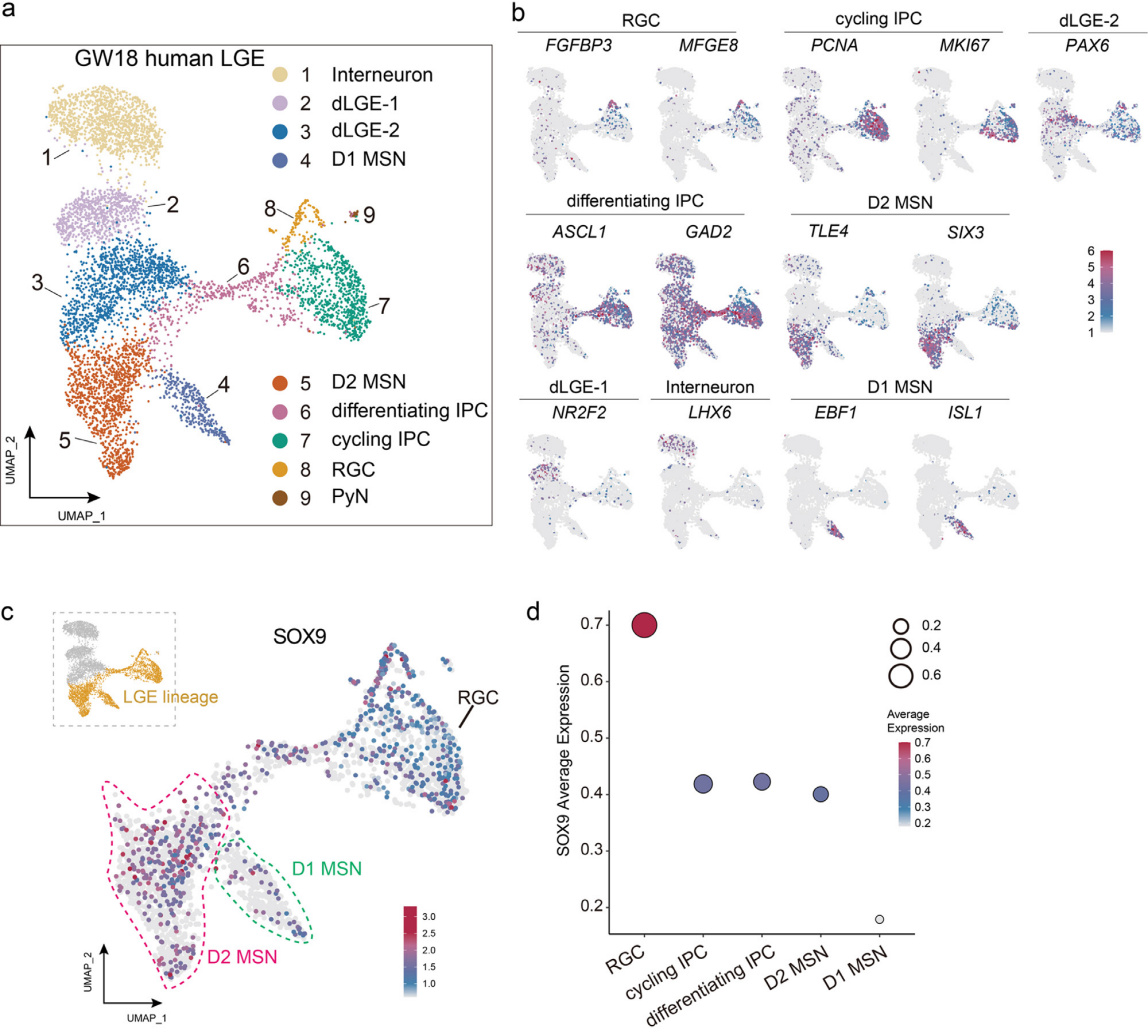
**The expression profile of *SOX9* in the LGE of human GW18 embryo**. (a) UMAP plot shows 9 clusters of human GW18 LGE colored by cell-type as identified by DEGs and typical marker genes. (b) UMAP plot shows the expression profiles of typical marker genes in different clusters. Each dot colored by gene expression level, represents an individual cell. (c) Violin plot shows relative expression of genes enriched in different cell types. Columns and rows represent typical genes and individual clusters, respectively, while color represents the gene expression level. (d) *SOX9* expression levels in LGE lineage cell clusters. UMAP plot shows *SOX9* expression level of the LGE lineage clusters, which were selected from the whole data (marked by yellow, top left). The dots inside the green and red dashed lines represent D1 and D2 MSNs, respectively. (e) *SOX9* expression levels in different clusters of human GW18 LGE lineage. Circle size represents the *SOX9*^+^ ratio among each cluster while the colors mean the average expression level of *SOX9*, in the LGE lineage clusters.

The expression of *Sox9* in striatal MSNs, especially in D2-MSNs, in mice has encouraged us to investigate whether this phenotype is specific to mice or conserved in humans during striatal development. To check this, we re-analyzed the scRNA-seq data of human fetal LGE at GW18 [23] (Fig. 3a). After quality control (Fig. S3a), unsupervised clustering and analysis of different gene expressions, these human LGE derived cells were divided into 9 clusters (Fig. 3a). Similar to the mouse data, canonical cell types of LGE, such as radial glia, IPC, D1-MSN, D2-MSN, dLGE cells, and interneurons, were identified. We also detected a subpopulation of dLGE cells that expressed *PAX6* [25] and cortical pyramidal neurons (Fig. 3b). Feather plots of specific genes and violin plots confirmed the classification of the LGE cells (Fig. 3b and Fig. S3b). We found that *SOX9* is expressed in radial glial cells, progenitor cells (including cycling and differentiating progenitor cells), and D2-MSNs (Fig. 3c). It should be noted that although the proportion of *SOX9^+^* cells in D2-MSNs was not very high compared to radial glial cells (Fig. 3d), the majority of the *SOX9^+^* cells in D2-MSNs had a relatively high expression level (Fig. 3c, 3d) suggesting that the expression pattern of *SOX9* in the LGE is conserved between humans and mice. This indicates that *Sox9* could be a specific marker for D2-MSNs and may regulate the development of post-mitotic D2-MSNs.

### 3.4 The expression of *Sox9* in the striatum is a dynamic process

**Fig. 4:**
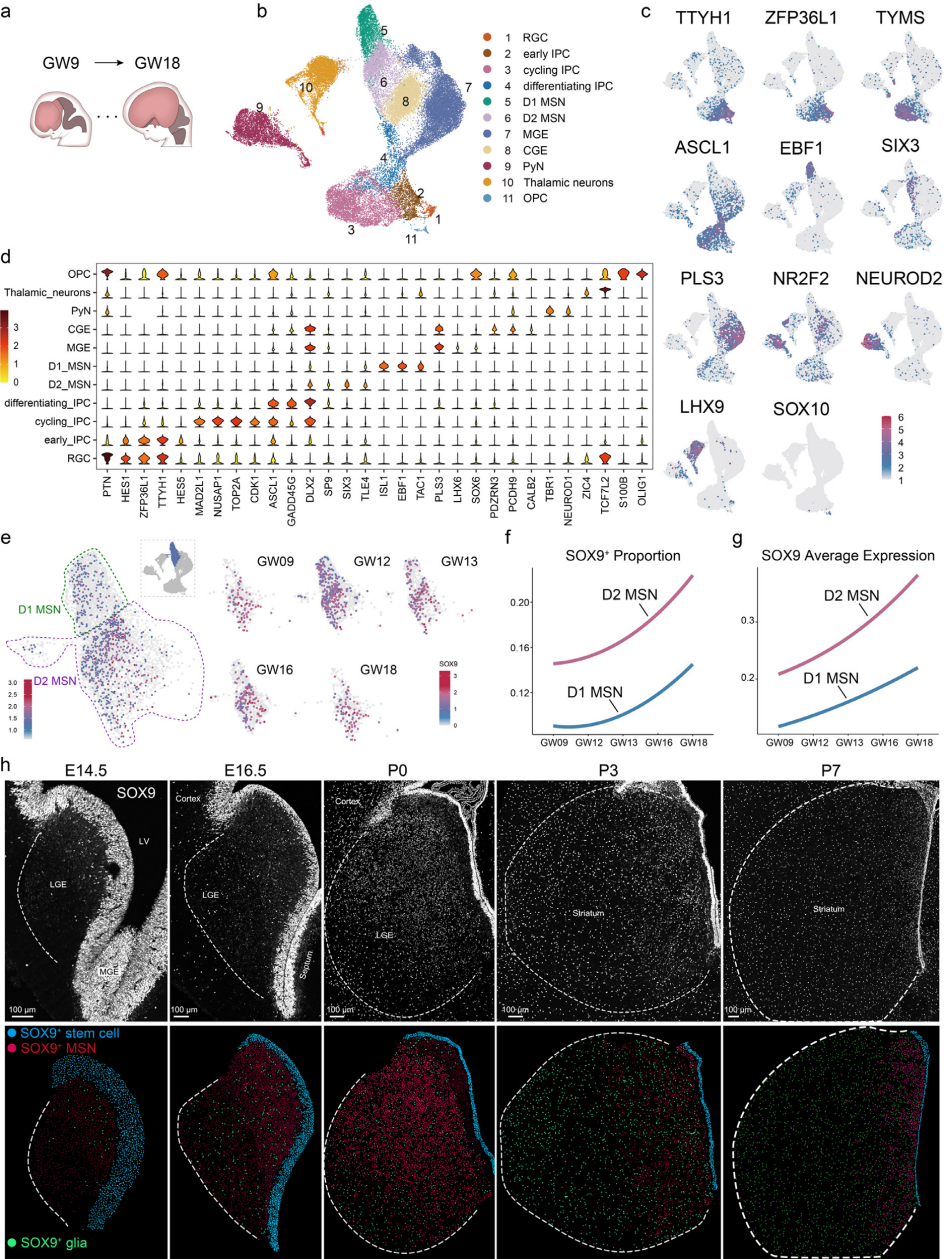
**Dynamic expression pattern of *Sox9* during LGE development.** (a) Summary schema of the samples used in the analysis of human GE development. (b) UMAP plot shows 11 clusters of GW9-GW18 human GE colored by cell-type as identified by DEGs and typical marker genes. (c) UMAP plot shows the expression profiles of typical marker genes in different clusters. Each dot colored by gene expression level, represents an individual cell. (d) Violin plot shows relative expression of genes enriched in different cell types. Columns and rows represent typical genes and individual clusters, respectively, while colors represent the gene expression level. (e) UMAP plot shows *SOX9* expression levels of MSN clusters selected from the whole data (marked by blue, top). The dots inside the green and red dashed lines represent D1 and D2 MSNs, respectively. The *SOX9* expression profiles at each time points are shown in the right panel. (f) Dynamic changes of *SOX9^+^* cell proportion and average expression level (g) in D1 and D2 MSNs during human brain development (GW9-GW18). (h) Top panel shows representative SOX9 immunostaining (white) of E14.5, E16.5, P0, P3, P7 mouse LGE. Dashed line indicates the striatum area. Schematics are shown in the bottom panel.

To further explore the expression pattern of *Sox9*, particularly the dynamic nature in human striatal development, we re-analyzed multiple scRNA-seq datasets from human ganglia eminences, including LGE, MGE, and caudal ganglionic eminence (CGE, Fig. 4a), dissected from GW 9 to 18 [23]. After quality control and filtering, unsupervised clustering was applied to collectively analyze the obtained 39,318 single cell transcriptional profiles from GW 9 to GW18 (Fig. S4a-c). Eleven clusters were identified and annotated using well-known cell type-specific markers (Fig. 4b, 4c). These clusters included radial glial cell (e.g. markers *HES1, ZFJP36L1*, and *TTYH1*), early IPC (e.g. markers *HES5, ASCL1*, and *HES1*), cycling IPC (e.g. markers *CDK1* and *TOP2A*), differentiating IPC (e.g. markers *DLX2* and *GADD45G*), D2-MSN (e.g. markers *SIX3* and *TLE4*), D1-MSN (e.g. markers *ISL1, EBF1*, and *TAC1*). In addition, numerous MGE derived cells (e.g. markers *PLS3* and *LHX6*), CGE derived cells (e.g. markers *NR2F2* and *CALB2*), thalamic neurons (e.g. markers *LHX9* and *TCL7L2*), pyramidal neurons (e.g. markers *TBR1* and *NEUROD2*) and OPC (e.g. markers *SOX10, S100B*, and *OLIG1*) were also involved (Fig. 4c, 4d). Strong expression of *SOX9* in MSNs, particularly in D2-MSNs, was observed at all stages (Fig. 4e).The ratio and average expression of *SOX9* in both D1 and D2-MSNs showed that the proportion of *SOX9*^+^ cells gradually increased in the human LGE from GW 9 to GW18 (Fig. 4e), consistently higher in D2-MSNs than in D1-MSNs (Fig. 4f). Moreover, the expression level of *SOX9* was also consistently higher in D2-MSNs, with a gradual increase in both subtypes during development (Fig. 4g). This pattern was mirrored in the mouse LGE, suggesting a biased expression pattern of *Sox9* in D2-MSNs during striatal development.

**Fig. 5:**
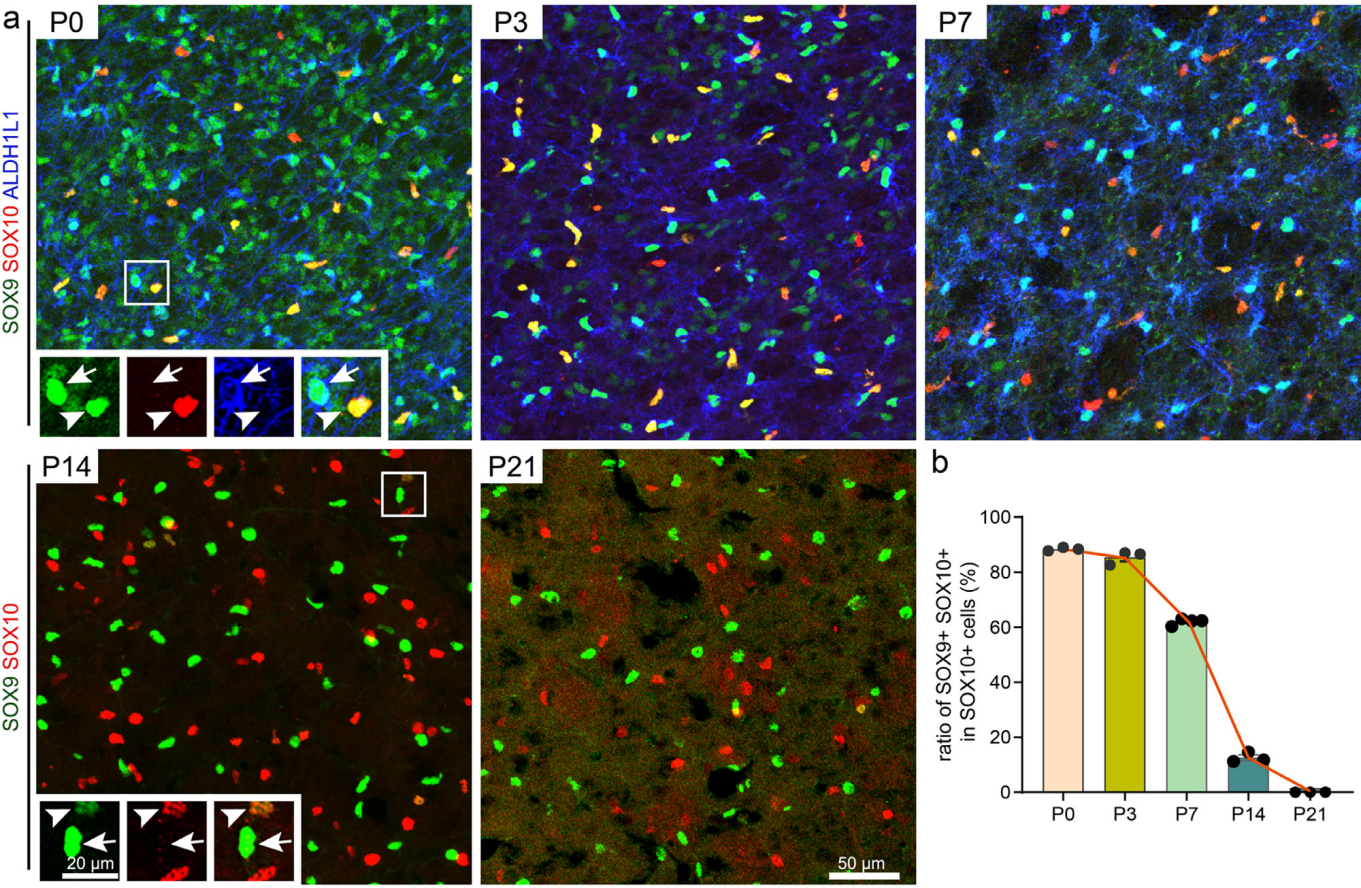
**Dynamic changes of SOX9 in the striatal oligodendrocyte development**. (a) Immunostaining images stained for SOX9 with SOX10 (P0-P21), and ALDH1L1 (P0-P7) in the striatum. Representative magnifications are shown in the inserted images, with arrows indicate astrocyte, arrowheads indicate oligodendrocyte. (b) Quantification of the ratios of SOX9^+^ SOX10^+^ in SOX10^+^ cells.

Immunostaining of SOX9 at various stages, from E14.5 to P7, confirmed this expression pattern in the mouse brain. At both E14.5 and E16.5, SOX9^high^ cells were prominent in the LGE VZ, while SOX9^low^ cells were widely distributed in the marginal zone (MZ), especially in the dorsal-medial part (Fig. 4h). Notably, these SOX9^low^ cells co-expressed BCL11B, indicating they are MSNs. This phenotype was also observed at P0, yielding the same result (Fig. 4h). It should be highlighted that the expression levels of individual SOX9^+^ striatum MSNs were comparable to those in the VZ or glial cells (Fig. 4h, middle panel). Interestingly, the number of striatal SOX9^+^ MSNs dramatically decreased by P3 and few cells were detectable by P7 (Fig. 4h and Fig. S4d), suggesting that *Sox9* is transiently expressed in MSNs, especially in D2-MSNs, during development, as most SOX9^+^ cells co-expressed SP9. Notably, the dynamic changes of the expression level of SOX9 in the striatum gradually increased from E14.5 to E16.5, peaked in the perinatal period and rapidly diminished after birth (Fig. 4h and Fig. S4d). However, SOX9^high^ cells became extensively distributed as striatal development progressed. Given that SOX9 is reported as an astrocyte marker, we speculated that the increased population of SOX9^high^ cells represented glial cells. At P0, the relative SOX9^high^ cells either expressed ALDH1L1, a pan-astrocyte marker [26], or were co-labeled with SOX10, a specific marker of oligodendrocytes (Fig. 5a), indicating that SOX9 is a pan-glial marker at this stage. Moreover, few SOX10^+^ cells without SOX9 expression were observed in the striatum at P0 (Fig. 5a, 5b). But the ratio of SOX10^+^SOX9^+^cells dramatically decreased at P7 and no SOX10^+^SOX9^+^ cells could be detected at P21 (Fig. 5a, 5b). This declining ratio of SOX9^+^SOX10^+^ cells among SOX9^high^ cells indicated that, similar to the transient expression pattern in MSNs, *Sox9* is temporarily expressed in OPCs and gradually becomes restricted to the astrocyte lineage during glial development. This observation is consistent with previous studies [[27], [28], [29]]. Taken together, these data suggest that *Sox9* is transiently expressed in D2-MSNs and OPCs, but constantly expressed by astrocytes.

### 3.5 *Sox9* is involved in the regulation of synaptogenesis in striatal MSNs

**Fig. 6:**
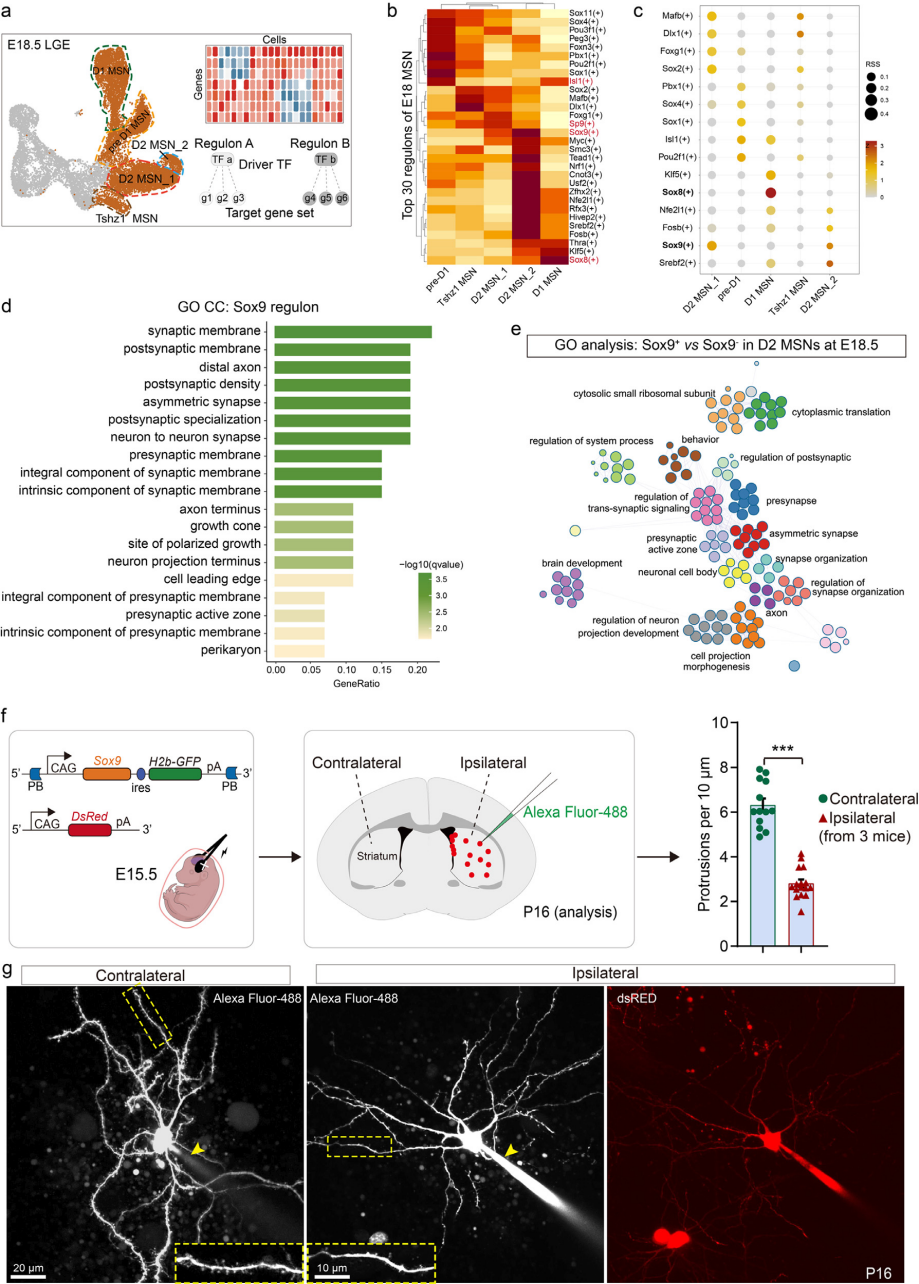
**The striatal *Sox9* expression is correlated with synaptogenesis.** (a) Schematics of the data selected and analysis workflow of SCENIC. (b) Heatmap shows the activities of the top 30 regulons in each cluster. (c) Dot plots show the selected regulon specificity scores among the top 30 regulons in each cluster. (d) GO analysis of genes in the Sox9 regulon network. (e) GO analysis of genes between the Sox9^+^ versus Sox9^−^ D2 MSNs at E18.5. (f) Schematic workflow overview of cell morphological reconstruction and quantification of dendritic spine number (g) Representative images of morphology of contralateral control cells and ipsilateral *Sox9* overexpression cells. Local magnification showed in the dashed frames. Arrow headers indicates glass electrodes filled with Alexa Fluor-488.

To investigate its role in MSNs, especially in D2 MSNs, we conducted single-cell regulatory network inference and clustering (SCENIC) analysis [19]. Focusing on MSNs and excluding the effects of Sox9^+^ stem cells, we extracted MSN clusters from the E18.5 mouse LGE scRNA-seq data (Fig. 6a). A total of 370 regulons were identified (Table S1). The top 30 regulons, based on AUC scores, were selected for analysis of transcription factor average activity. Each cluster displayed specific active regulons, such as Sox11(+) and Sox1(+) regulons were active in pre-D1 cluster, while Sox2(+), Mafb(+), and Dlx1(+) regulons in Tshz1^+^ MSNs showed strong activities; the D1-MSN cluster exhibited high activity of the Sox8(+) regulon (Fig. 6b), and the D2-MSN cluster showed strong activities of the Sp9(+) and Sox9(+) regulons (Fig. 6b).

**Fig. 7:**
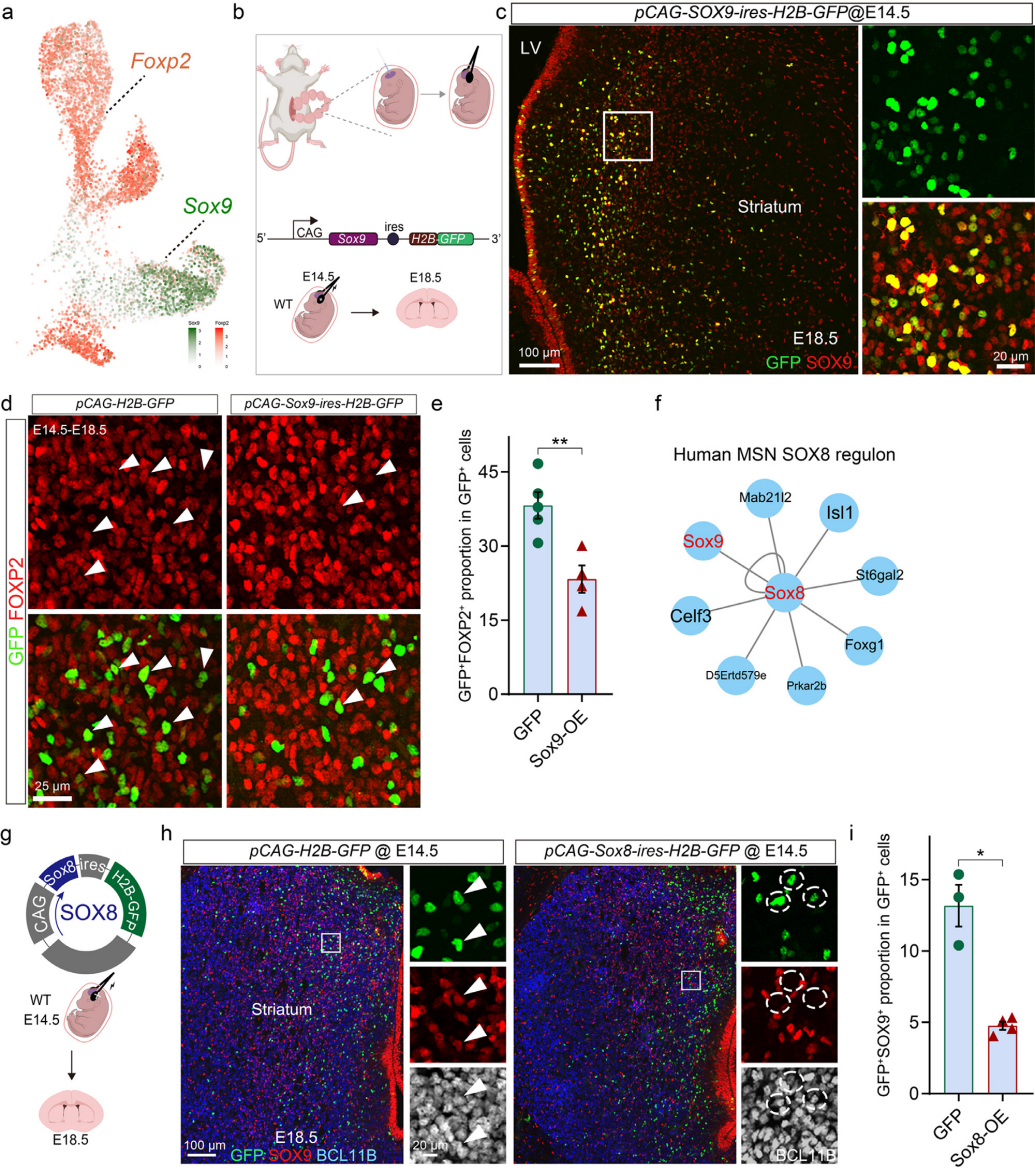
***Foxp2* is a synaptogenesis target of *Sox9* that is repressed by *Sox8*.** (a) Dual gene plot shows the expression profiles of *Foxp2* and *Sox9* in MSNs of E18.5 scRNA-seq. (b) Schematic workflow of IUE and strategy of *Sox9* overexpression in the LGE. (c) Representative image of SOX9 and GFP immunostaining after overexpression in the LGE for 4 days (E14.5-E18.5). (d) Representative images of SOX9 and FOXP2 immunostaining in both control (*pCAG-H2B-GFP*) and *Sox9* overexpression group. (e) Quantification of the ratio of GFP^+^ FOXP2^+^ cells in GFP^+^ cells in the two groups in (d). (f) Predicted Sox8(+) regulon network derived from the analysis of human MSNs in the GW9-GW18 dataset. (g) Schematic workflow of *Sox8* overexpression in the LGE. (h) Representative images of SOX8 and SOX9 immunostaining in both control (*pCAG-H2B-GFP*) and *Sox8* overexpression group. (i) Quantification of the ratio of GFP^+^ SOX9^+^ cells in GFP^+^ cells in the LGE of the two groups in (h).

Regulon Specificity Scores (RSS) in each cluster further confirmed the preference of the Sox9(+) regulon in D2-MSN clusters (Fig. 6c), while the Sox8(+) regulon was specifically active in D1-MSNs (Fig. 6c). Mapping the regulons onto the UMAP of scRNA-seq data revealed rapid downregulation of Dlx1(+) regulon activity in both D1-MSNs and D2-MSNs. The Sp9(+) regulon displayed sustained activity in D2-MSNs, but was gradually downregulated in D1-MSNs (Fig. S4e), consistent with its known expression pattern [21]. Similarly, Sox8(+) and Sox9(+) regulons showed distinct activities in D1-MSNs and D2-MSNs, respectively [30] (Fig. S4e). Notably, the Sp9(+) regulon was linked to several D2-MSN specific genes, like *Six3* and *Adora2a*, and *Sox9* is a putative target of *Sp9* in regulating D2-MSN development [4,6] (Fig. S4f), providing further evidence for the correlation between *Sox9* and D2-MSNs. The Sox9(+) regulon comprised 29 potential target genes (Fig. S4g) that were primarily enriched in synaptic development (Fig. 6d), indicating a strong association between *Sox9* expression and synaptogenesis. To further investigate the role of *Sox9* in MSNs, we compared the DEGs between *Sox9*^+^ and *Sox9*^−^ cells in D2-MSNs at E18.5 followed by GO analysis. The analysis revealed that most of the GO terms were related to axon and synapse development signaling, such as the regulation of postsynaptic, presynapse, and synapse organization (Fig. 6e). To confirm this, we performed an in vivo analysis of synaptogenesis. Mix vectors of *Sox9* overexpression and *dsRed* reporter were electroporated into the LGE at E15.5 (Fig. 6f). The overexpression efficiency was verified by co-labeling GFP cells with SOX9 (Fig. 7c). Brains were collected and sectioned at P16. GFP^+^ dsRed^+^ cells in the ipsilateral striatum were patched and filled with Alexa Fluor-488, which was synchronously detected using a two-photon laser scanning microscopy to visualize the morphology. Cells in the contralateral striatum without GFP or dsRed expression were randomly chosen as controls (Fig. 6f). After morphological reconstruction, control cells exhibited numerous protuberances around the dendrites. However, relatively smooth dendrites were identified after Sox9 overexpression (Fig. 6g). Quantification of spines showed that the spine number was significantly reduced in Sox9 overexpression cells (Fig. 6f). Overall, these findings suggest that the primary function of *Sox9* in MSNs is mainly related to synaptic development.

### 3.6 *Sox9* regulates synaptic development via repressing *Foxp2*

It has been reported that *Sox9* binds to the promoter of *Foxp2*, regulating its expression [31]. Additionally, *Foxp2* is shown to play a role in synaptic development [32]. In the scRNA-seq data, we found that *Foxp2* was primarily expressed in D1-MSNs and showed a complementarity with *Sox9* in the striatum suggesting mutual repulsion between the two factors (Fig. 7a). Therefore, we hypothesized that *Sox9* regulates synaptic development through *Foxp2*. To test this, we overexpressed *Sox9*, via the CAG promoter, in the LGE at E14.5 and analyzed *Foxp2* expression at E18.5, using nuclear H2B-GFP as a reporter. Vectors containing only the H2B-GFP reporter gene served as the control (Fig. 7b). It seems that more GFP^+^ cells were located at SVZ following *Sox9* overexpression, possibly because the strong expression of Sox9 maintains the stemness of progenitor cells. However, GFP^+^ cells of both groups could disperse into the MZ where we performed quantification as we focus on the function of Sox9 in post-mitotic MSNs.

We discovered that both the ratio of FOXP2^+^GFP^+^ cells wtihin the total GFP^+^ cell population in the MZ and the expression level of FOXP2 in GFP^+^ cells were significantly reduced following *Sox9* overexpression (Fig. 7d, 7e) indicating that *Sox9* inhibits striatal *Foxp2* during striatal development.

### 3.7 *Sox8* regulates the selective expression pattern of *Sox9* in D2-MSNs

Interestingly, a previous study showed that *Sox8*, a homolog of *Sox9*, is specifically expressed in D1-MSNs [30]. Consistently, we observed that *Sox8* and *Sox9* showed complementary expression patterns in the MSN according to scRNA-seq data (Fig. S4e). This phenotype is conserved across mouse and human datasets. Members of the SOXE family often show mutual regulation of each other [[33], [34], [35], [36]]. Furthermore, SCENIC analysis of human scRNA-seq data indicated that *SOX9* might be a latent downstream target of *SOX8* within the identified SOX8(+) regulon (Fig. 7f). Thus, we speculate that the weak expression level of *Sox9* in D1-MSNs could be attributed, at least in part, to the inhibitory effect of *Sox8* on *Sox9* expression. To explore this hypothesis, we overexpressed *Sox8* in the LGE and assessed the impact on *Sox9* expression. Similar to the overexpression of *Sox9*, vectors were *in utero* electroporated into the LGE at E14.5 and analyzed at E18.5 (Fig. 7g). We found that the ratio of GFP^+^SOX9^+^ cells in GFP^+^ cells in the MZ was significantly decreased after overexpression of *Sox8* (Fig. 7h, 7i), supporting our hypothesis. Taken together, these findings suggest that *Sox8* in the striatum represses *Sox9* expression, leading to the restriction of *Sox9* to D2-MSNs.

## 4 Discussion

Striatum, the largest nucleus in the basal ganglia of the forebrain, is associated with specific mental diseases and cognitive deficits when underdeveloped [1]. Understanding neurogenesis and synaptogenesis is crucial for enhancing therapeutic outcomes for related mental diseases, such as Parkinson’s disease and autism. In this study, we have identified that *Sox9*, traditionally known as a glial marker, is also uniquely expressed in striatal MSNs, especially in D2-MSNs. This suggests an important role for *Sox9* in D2-MSN development. Additionally, we observed that *Sox9* expression in LGE MSNs follows a dynamic pattern, starting at low levels during early striatal development, peaking during the perinatal period, and rapidly diminishing after birth. Notably, this dynamic expression pattern of *Sox9* is conserved across both mice and humans. Gene regulatory network analysis indicates that targeted gene activity of *Sox9* is associated with D2-MSNs and plays a role in synaptogenesis. Importantly, we demonstrate that *Sox9* regulates synaptogenesis by inhibiting *Foxp2* expression. Furthermore, *Sox8* has been identified as an upstream regulator of *Sox9*, contributing, at least partially, to the biased expression pattern of *Sox9* in D2-MSNs. Collectively, our findings reveal a previously unidentified striatum-specific function of *Sox9*, providing insights into striatal development and related diseases.

Previous reports has indicated that *Sox9* is expressed in radial glial stem cells or macroglia, playing distinct roles in their development [[14], [15], [16]]. Our scRNA-seq data analysis revealed that *Sox9* is also expressed in striatal projection neurons. Further analysis showed a preference for *Sox9* in D2-MSNs in terms of both proportion and expression levels. Although a small proportion of D1-MSNs in the scRNA-seq data exhibited *Sox9* expression, the ratio of SOX9^+^ ISL1^+^ cells identified through immunostaining was significantly smaller. This discrepancy may arise from the relatively high sensitivity of sequencing, capable of detecting low levels of *Sox9* mRNA that may not translate into detectable protein levels. This is consistent with our observations, where the average expression level of *Sox9* in D1-MSNs was very low, resulting in a smaller observed co-expression ratio of SOX9^+^ ISL1^+^ in our experimental analysis. Similarly, we identified a subpopulation of *Sox9*^+^ cells among intermediate progenitor cells, though their protein levels were very low, likely for the same reason.

## 5 Conclusion

Based on these results, we conclude that *Sox9* is specifically expressed in D2-MSNs. It is noteworthy that during the early stages of striatal development, *Sox9* levels are very low, peaking during the perinatal period and rapidly downregulated after birth. This peak expression coincides with the transition from neuron generation to maturation, primarily involving synaptogenesis and integration [32]. Indeed, the analysis of Sox9(+) regulons and DEGs between *Sox9^+^* and *Sox9^−^* MSNs confirms that *Sox9* activity is strongly linked to synaptogenesis. Although this speculation has not been validated in a transgenic model, we believe to be accurate, as neurogenesis is nearly complete by the time *Sox9* is highly expressed.

The *Sox* gene family, comprising numerous members divided into eight subfamilies A–H, based on the HMG box amino acid sequence [37], includes *Sox8, Sox9*, and *Sox10* in the SoxE subclass in most vertebrates [38]. SoxE proteins regulate various aspects of both the peripheral and central nervous systems, overseeing stemness, survival, specification, and glial differentiation [28,39,40]. The functions of SoxE proteins often overlap and are redundant [33,36,41]. In this study, we discovered that *Sox8* is activated in D1-MSNs and complements *Sox9* in striatal MSNs suggesting possible mutual repression. Indeed, the overexpression of *Sox8* inhibited *Sox9* expression during LGE development, implying that the low expression level of *Sox9* in D1-MSNs may be due to the repression exerted by SOX8 on *Sox9*. This interaction likely begins at the progenitor stage and becomes evident at the post-mitotic stage. In considering the downstream regulators of *Sox9*, we identified *Foxp2* as a strong candidate significantly enhancing striatal synapse formation upon overexpression. In addition to *Foxp2, Mef2c*, showing a positive correlation with *Sox9* expression in the scRNA-seq data DEGs and known to interact with *Foxp2* in synapse development [32], also emerges as a strong candidate. This hypothesis warrants further investigation. Altogether, our study enhances our understanding of the function of *Sox9* in synaptic development in the striatum and may inform the treatment of related disorders.

## CRediT authorship contribution statement

Z.X., X.S., R.G. conceptualized the project. X.S., X.L., R.G. analyzed the data. X.S. R.G. performed most of the experiments. X.P., H.Y., K.W. performed the morphological reconstruction. T.Y., X.X., L.G. quantified the data. W.L. revised the manuscript. X.S., Z.X. designed the experiments, produced all figures and wrote and revised the manuscript with input from all authors.

## Declaration of competing interest

The authors declare that they have no conflicts of interest in this work.

## Appendix Supplementary materials

Image, application 1

Image, application 2
