# Supplementary material for: The SoxE factor Sox9 is selectively expressed in indirect pathway striatal projection neurons and regulates synaptogenesis
Source: Fundam Res. 2024 Apr 2;6(2):906–18. doi: 10.1016/j.fmre.2024.02.019 (PMC13069653; doi:10.1016/j.fmre.2024.02.019)
Supplement: Supplementary file 1 [file mmc1.pdf]

**Supplementary Figures S1 to S4**

**Supplementary Table S1**

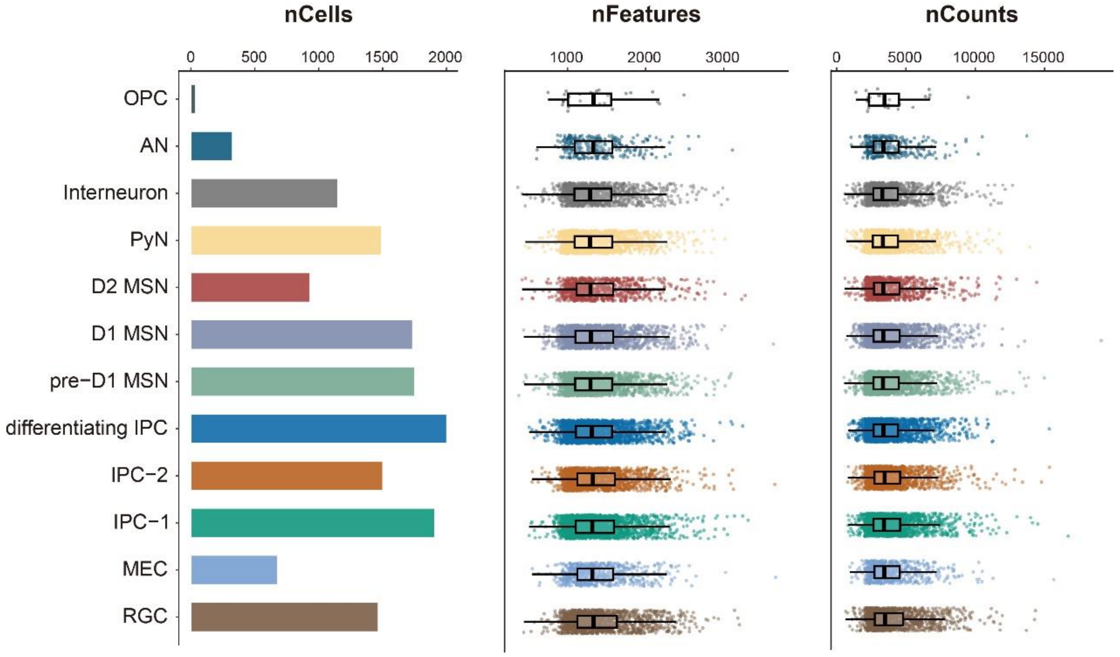


**Supplemental Figure 1. Quality control of E14.5 mouse LGE scRNA-seq.**

All cells used in the clustering analysis of scRNA-seq of E14.5 mouse embryo are illustrated. The number of cells in 12 clusters is visualized with histogram (left part). The gene number and sequencing count number of each cell are shown by clusters, respectively (middle and right part). Each dot represents a single cell and is colored according to its cluster.

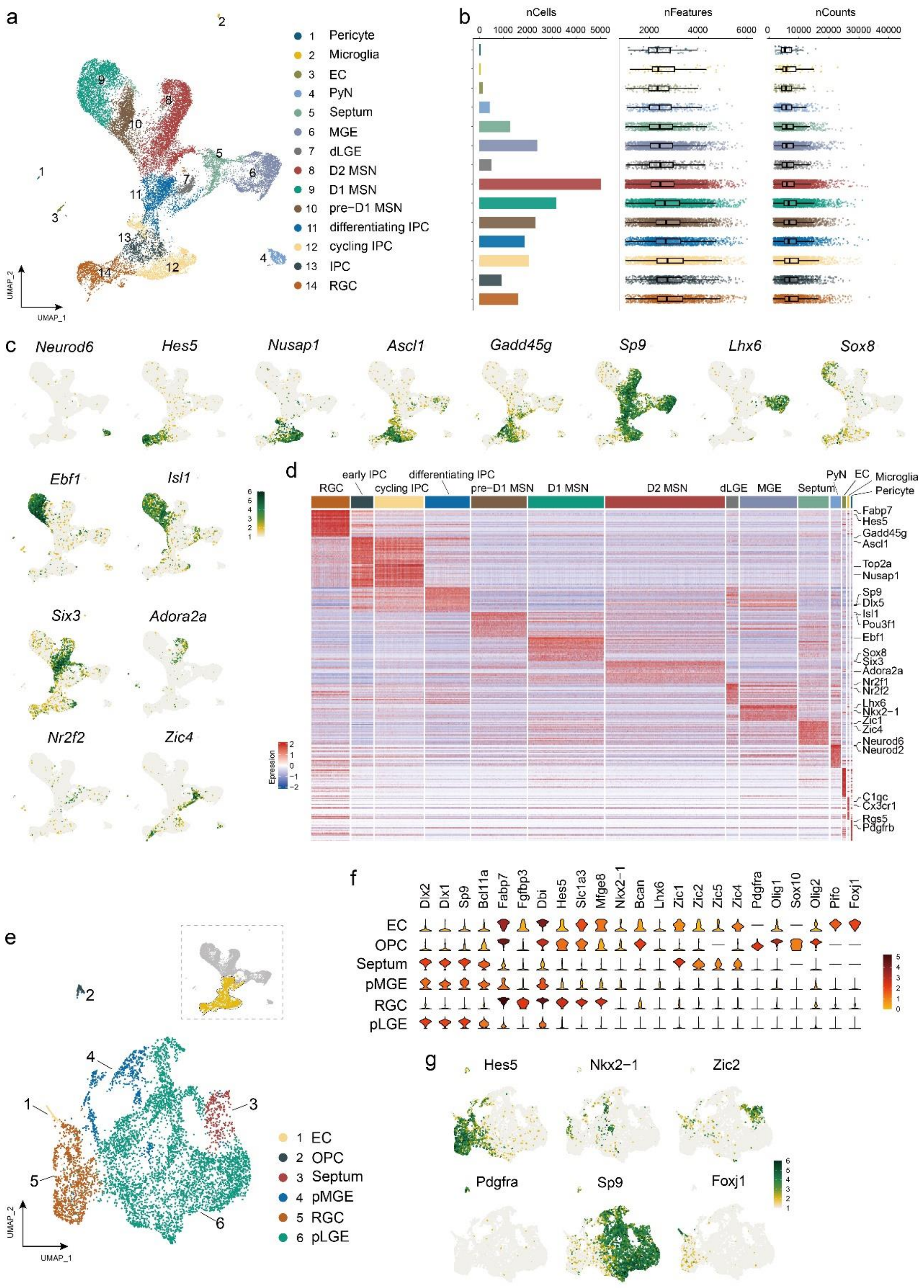
a
1 Pericyte
2 Microglia
3 EC
4 PyN
5 Septum
6 MGE
7 dLGE
8 D2 MSN
9 D1 MSN
10 pre-D1 MSN
11 differentiating IPC
12 cycling IPC
13 IPC
14 RGC
UMAP_1
UMAP_2
b
nCells
nFeatures
nCounts
c
Neurod6
Hes5
Nusap1
Ascl1
Gadd45g
Sp9
Lhx6
Sox8
Ebf1
Isl1
Six3
Adora2a
Nr2f2
Zic4
d
RGC
early IPC
cycling IPC
differentiating IPC
pre-D1 MSN
D1 MSN
D2 MSN
dLGE
MGE
Septum
PyN
EC
Microglia
Pericyte
Fabp7
Hes5
Gadd45g
Ascl1
Top2a
Nusap1
Sp9
Dlx5
Isl1
Pou3f1
Ebf1
Sox8
Six3
Adora2a
Nr2f1
Nr2f2
Lhx6
Nkx2-1
Zic1
Zic4
Neurod6
Neurod2
C1qc
Cx3cr1
Rgs5
Pdgfrb
Epression
e
1 EC
2 OPC
3 Septum
4 pMGE
5 RGC
6 pLGE
UMAP_1
UMAP_2
f
Dlx2
Dlx1
Sp9
Bcl11a
Fabp7
Fgfbp3
Dbi
Hes5
Slc1a3
Mfge8
Nkx2-1
Bcan
Lhx6
Zic1
Zic2
Zic5
Zic4
Pdgfra
Olig1
Sox10
Olig2
Pifo
Foxj1
EC
OPC
Septum
pMGE
RGC
pLGE
g
Hes5
Nkx2-1
Zic2
Pdgfra
Sp9
Foxj1

**Supplemental Figure 2. Clustering of mouse E18.5 ventral telencephalon scRNA-seq**

(**a**) UMAP plot displays 14 clusters of E18.5 mouse ventral telencephalon, colored by cell type identified by DEGs and typical marker genes.

(**b**) Quality control of mouse E18.5 ventral telencephalon scRNA-seq.

(**c**) UMAP plot shows the expression profiles of typical marker genes in 14 clusters. Each dot, colored by gene expression level, represents an individual cell.

(**d**) Heatmap demonstrates the relative expression of genes enriched in the 14 different cell types. Typical cell-type marker genes are labeled.

(**e**) UMAP plot illustrates the re-clustering for the progenitor cells of E18.5 mouse ventral telencephalon, colored by cell type identified by DEGs and typical marker genes.

(**f**) Violin plot shows relative expression of genes enriched in different cell types. Columns and rows represent typical genes and individual clusters, while color represents the gene expression level.

(**g**) UMAP plot shows the expression profiles of typical marker genes in 6 progenitor cell clusters. Each dot, colored by gene expression level, represents an individual cell.

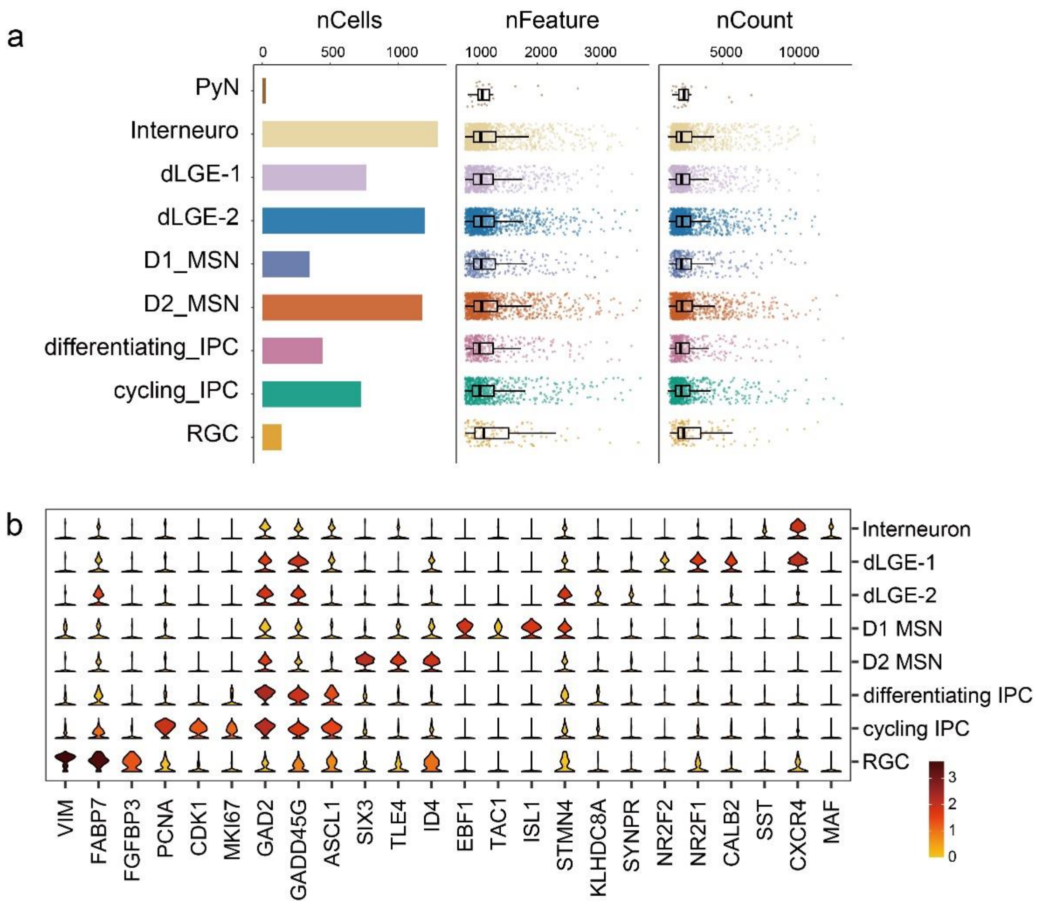


**Supplemental Figure 3. Quality control of GW18 human LGE scRNA-seq.**

**(a)** All cells used in the clustering analysis of scRNA-seq of GW18 human LGE was illustrated. The number of cells in 9 clusters were visualized with histogram (left part). The gene number and sequencing count number of each cells were shown by clusters, respectively (middle and right part). Each dot represents a single cell, colored according to its assigned cluster.

**(b)** Violin plot shows relative expression of genes enriched in different cell types. Columns and rows represent typical genes and individual clusters, respectively, while color represents the gene expression level.

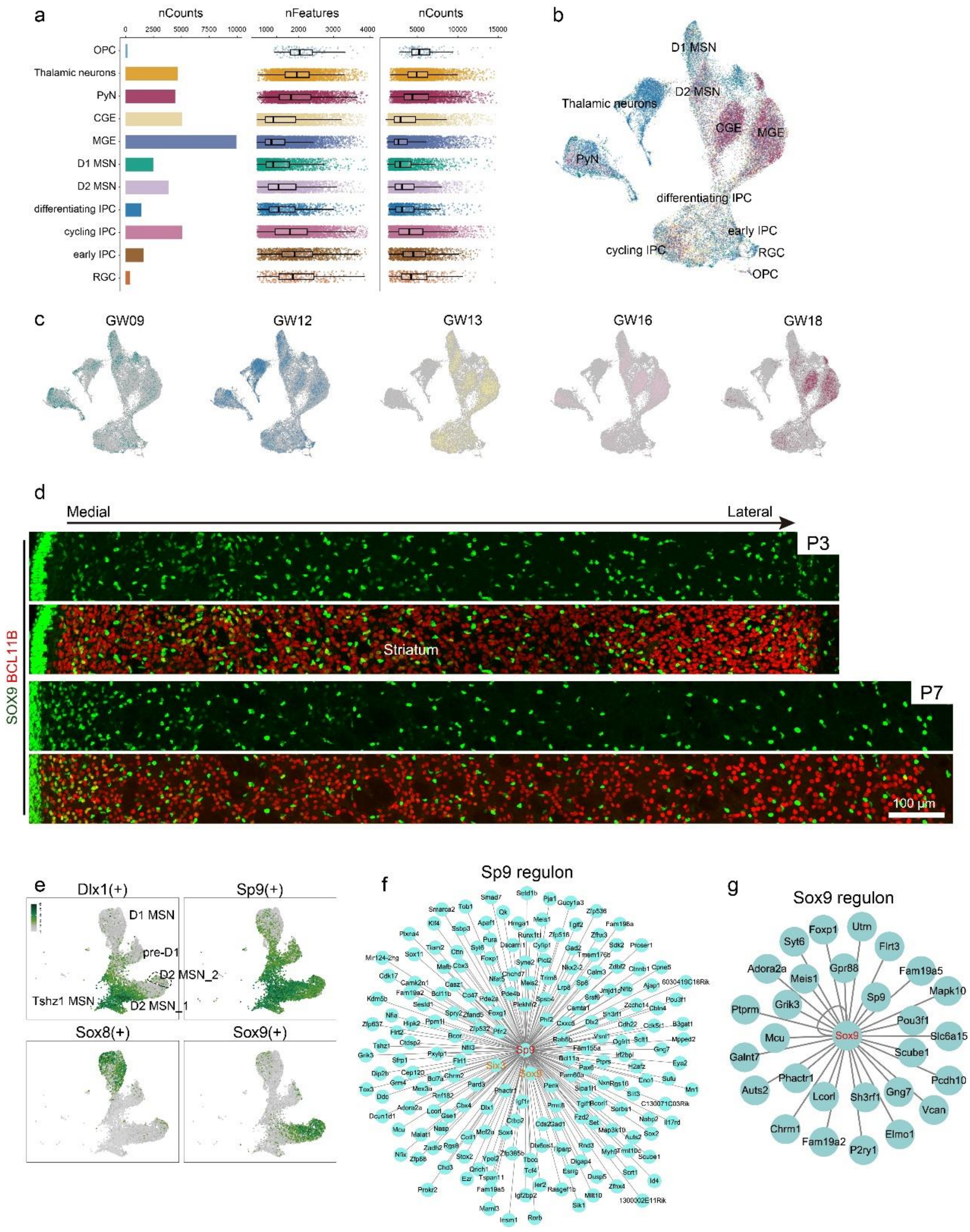


**Supplemental Figure 4. Dynamic changes of SOX9 in the developing striatum.**

(**a**) Quality control of the scRNA-seq dataset of human ganglia eminences collected from GW09 to GW18.

(**b**) UMAP plot shows GW09-18 human ganglia eminence scRNA-seq dataset, colored based on different timepoints of development (GW09-18).

(**c**) UMAP plot shows GW09, GW12, GW13, GW16 and GW18 human ganglia eminence cells distribution in the clusters.

(**d**) Immunostaining for SOX9 and BCL11B from medial to lateral, at P3 and P7 striatum.

(**e**) UMAP plots show the selected Dlx1(+), Sp9(+), Sox8(+), and Sox9(+) regulon activity in MSNs.

(**f-g**) Predicted Sp9(+) and Sox9(+) regulon (**e**) network**.**

**Supplementary Table S1.** Total SCENIC regulons in the scRNA-seq data of E18.5 LGE.
